# Fatal Errors

**Published:** 1881-06

**Authors:** Ad. Lippe

**Affiliations:** Philadelphia


### FATAL ERRORS.

AD. LIPPE, M.D., PHILADELPHIA.

It is a fatal error to claim that the law of the similars is not a universal law governing the therapeutics of the homœopathic school of medicine; it is still a more fatal error to say that the law of the similars and the "law of the contraries" are not antagonistic; that they are supplemental, one supplying the deficiency of the other. These fatal errors have been exposed before; they are such gross errors that it is really painful to combat them again; but, as we have time and again asked the error worshipers to "illustrate," without a response, we must rejoice to find an illustration at last. This fatal error is uttered first by Joseph Kidd, M.D., and was by him published above his signature, April 15th, 1881, in *The London Times.* He is there and then endorsed by R. E. Dudgeon, M.D., who says "*The partisans of the reformed system only claim for homœopathy that it [the law of the similars] is the best rule for the selection of the drugs, but they admit that there may be cases for which a homœopathic remedy can not be found, and where some other means must be had recourse to.*"

A fatal error indeed! The true healer is always guided by the law of the similars and while he knows that a law must either admit of a universal application or if that is not practicable, that it ceases to be a law and becomes only a good or best rule, has, by accepting the honorable name of homœopathist, thereby acknowledged that the law of the similars is a universal law in therapeutics; on the other side, it is the prerogative of the eclectic school to accept every method and means that may be considered useful in the treatment of the sick, which prerogative Dr. Dudgeon illogically claims to belong to homœopathy. But we offer no more suggestions or arguments against this monstrous absurdity, this fatal error; we see it finally illustrated. Dr. Kidd, a professed homœopathic physician, became imbued with the idea that he was much wiser than was Hahnemann: failing to follow the master's example and failing to make the experiment successfully he first abandoned the potencies after setting aside the law of the similars, and, giving up his compass and rudder, was left to the winds and the waves; where did this professing homœopath drift? He writes to Dr. Quain, the allopathic physician called in consultation in the case of the late Lord Beaconsfield,

"Dear Sir,—I have to thank you for your communication. In reply, I beg to say that I am not treating Lord Beaconsfield homœopathically. I beg further to assure you that every direction and prescription of yours will be faithfully carried out by me. Believe me, yours very truly, J. Kidd.

Here we have, *at last*, an illustration. A grave case arises: a noble lord sickens, a professed homœopath abandons the only universal law of cure and applies an opposite law. Dr. K. believed that two laws would not antagonize!! that they could be supplemental, one supplying the deficiency of the other. Believing in such monstrous absurdities, he could not but come down to the final humiliating position of offering himself to Dr. Quain, to become his humblest of all servants—Dr. Quain's man Friday. Can a deficient law be supplanted by another deficient law? What was the result of the abandonment of homœopathy? A humiliating, nay, a disgraceful surrender to the allopathic school; and it proved to be of no benefit to the help seeking Lord B. A true healer will forever hold fast to his principles and the graver the case the more persistently will he apply himself to the best application of the law and principles governing the school to which he professes and does belong. Eclecticism is not homœopathy and never will take its place among the medical methods, save to be ridiculed by all honest practitioners of medicine, be they homœopathists or allopathists. The illustration, given by the would be perverters of homœopathy into ridiculous eclecticism, illustrates the fallacy of and the disgrace into which their propositions must by logical necessity, and in reality did, lead. Every honest healer must thank these men for having *at last* appeared on the witness-stand before the final medical tribunal and the common sense of all thinking men, and have testified. May they be rewarded.

